# Stannous Chloride-Modified Glass Substrates for Biomolecule Immobilization: Development of Label-Free Interferometric Sensor Chips for Highly Sensitive Detection of Aflatoxin B1 in Corn

**DOI:** 10.3390/bios14110531

**Published:** 2024-11-03

**Authors:** Alexey V. Orlov, Maria O. Zolotova, Denis O. Novichikhin, Nikolai A. Belyakov, Svetlana G. Protasova, Petr I. Nikitin, Artem V. Sinolits

**Affiliations:** 1 Prokhorov General Physics Institute of the Russian Academy of Sciences, 38 Vavilov Street, 119991 Moscow, Russia; alexey.orlov@kapella.gpi.ru (A.V.O.); zolotovamaria333@gmail.com (M.O.Z.); nammen@yandex.ru (D.O.N.); whitenik.bel@yandex.ru (N.A.B.); sinolits@geokhi.ru (A.V.S.); 2 Vernadsky Institute of Geochemistry and Analytical Chemistry, Russian Academy of Sciences, Kosygin Str. 19, 119991 Moscow, Russia; 3 Moscow Center for Advanced Studies, Kulakova Str. 20, 123592 Moscow, Russia; 4 Osipyan Institute of Solid State Physics, Russian Academy of Sciences, 142432 Chernogolovka, Russia; sveta@issp.ac.ru

**Keywords:** chlorostannate, surface modification, biofunctionalization, protein immobilization, micotoxins, spectral-phase interferometry, surface plasmon resonance, real-time sensor

## Abstract

This study presents the development of stannous chloride (SnCl_2_)-modified glass substrates for biomolecule immobilization and their application in fabricating sensor chips for label-free interferometric biosensors. The glass modification process was optimized, identifying a 5% SnCl_2_ concentration, a 45 min reaction time, and a 150 °C drying temperature as conditions for efficient protein immobilization. Based on the SnCl_2_-modified glass substrates and label-free spectral-phase interferometry, a biosensor was developed for the detection of aflatoxin B1 (AFB1)—a highly toxic and carcinogenic contaminant in agricultural products. The biosensor realizes a competitive immunoassay of a remarkable detection limit as low as 26 pg/mL of AFB1, and a five-order dynamic range. The biosensor performance was validated using real corn flour samples contaminated with *Aspergillus flavus*. The proposed approach not only provides a powerful tool for AFB1 detection for food safety monitoring but also demonstrates the potential of SnCl_2_-modified substrates as a versatile platform for the development of next-generation biosensors.

## 1. Introduction

The highly sensitive determination of aflatoxin B1 (AFB1) is of critical importance due to the extreme toxicity and widespread occurrence of the substance in various agricultural products [1,2,3,4]. AFB1 is one of the most dangerous known natural carcinogens [5,6], resistant to thermal processing [7], capable of causing liver damage [8], and associated with the development of hepatocellular carcinoma [9]. This toxin is produced by strains of *Aspergillus* molds, which contaminate food products [10,11,12], in particular, grains (such as corn and rice), nuts (especially peanuts), seeds, spices, and dried fruits, as well as animal feed [13,14] and animal products (if the animals consume contaminated feed) [15,16].

Traditional methods for AFB1 determination, based on high-performance liquid chromatography–mass spectrometry (LC-MS), are widely used due to their high sensitivity and specificity [17,18]. However, LC-MS approaches generally feature long analysis times, complex sample preparation, and expensive equipment [19,20]. Lately, numerous immunochemical methods have been proposed for faster and more convenient AFB1 detection [21,22,23,24,25]. These include various ELISA-based techniques and a multitude of electrochemical, chemiluminescent, and lateral flow assays.

Label-free optical methods [26,27] represent a promising immunoanalytical approach to AFB1 detection, and surface plasmon resonance (SPR) [28,29,30,31] is among the most popular ones. SPR-based methods offer real-time monitoring of biomolecular interactions and high sensitivity (with phase SPR demonstrating ultra-high sensitivity [32]). However, they require sophisticated equipment and expensive sensor chips with thin gold films precisely deposited on the surface [33,34]. In recent years, alternative label-free methods have been actively developed, in particular, those involving interference and utilizing biosensors with transparent glass surfaces, such as reflectometric interference spectroscopy [35], biolayer interferometry (BLI) [36,37], spectral-phase interferometry (SPI) [38,39], and spectral-correlation interferometry (SCI) [40,41].

One of the key factors for the performance of label-free interferometric immunosensors is the development of the sensor chip, which involves the modification of optically transparent glass substrates by proper functional groups to allow for the immobilization of recognition biomolecules such as antibodies [42,43,44]. Traditional techniques use covalent chemistry, with the carbodiimide method being especially popular [45]. In this method, carboxyl groups on the surface are activated, followed by the formation of a stable amide bond between the surface and antibody. Although such covalent bonds ensure the strong and stable immobilization of antibodies, these methods need chemically unstable reagents and, consequently, suffer from reproducibility issues [46,47].

Here, we have developed an alternative approach to the biofunctionalization of glass substrates, specifically, the modification of the surface with SnCl_2_ and subsequent one-step biomolecule immobilization. Notably, SnCl_2_-modified inorganic materials are promising for immobilizing thiol- and disulfide-containing biomolecules, such as cysteine-containing proteins, due to the strong bond between tin and sulfur atoms. Recently, SnCl_2_-modified porous silica [48] and silica-coated magnetite nanoparticles [49] were used for the immobilization of enzymes and antibodies, respectively. However, methods for using SnCl_2_-modified inorganic substrates for sensor chips are still to be developed.

In this work, SnCl_2_-modified glass substrates were developed, characterized, and optimized for the fabrication of sensor chips. The chips were then successfully tested for analytical applications using the label-free SPI. We demonstrate the efficiency of these chips for a competitive immunoassay for AFB1 detection with an SPI biosensor. Attractive analytical characteristics were obtained, e.g., a detection limit of 26 pg/mL and a five-order dynamic range of concentration. The proposed concept of biofunctionalized SnCl_2_-modified glass substrates can be used for a wide range of sensor chips in different biosensors, including those based on colored, fluorescent, chemiluminescent, or plasmonic labels.

## 2. Materials and Methods

### 2.1. Reagents

The following reagents were used in the experiments: isopropanol (Sigma-Aldrich, St. Louis, MO, USA), sulfuric acid (Merck, Darmstadt, Germany), hydrogen peroxide (30%, Sigma-Aldrich, St. Louis, MO, USA), tin(II) chloride dihydrate (Alfa Aesar, Ward Hill, MA, USA), phosphate-buffered saline tablets (PBS, Thermo Fisher Scientific, Waltham, MA, USA), bovine serum albumin (BSA, Sigma-Aldrich, St. Louis, MO, USA), conjugates of ovalbumin with aflatoxin B1 (OVA-AFB1), and corn flour samples infected with Aspergillus flavus and positive calibrators (DTS Biotech Ltd., Pushchino, Russia). All reagents were of analytical grade and were used without additional purification. The “pirranha” solution was prepared by mixing 10 mL H_2_O_2_ and 30 mL H_2_SO_4_ immediately before use. Deionized water (Millipore, Temecula, CA, USA) was used for all cleaning and rinsing procedures.

### 2.2. Purification of Glass Substrates

The glass substrates, which were microscope cover glasses (22 × 22 mm), were first washed with isopropanol for 15 min, then dried and treated with “pirranha” solution for 1 h in a polytetrafluoroethylene container. After that, the glass substrates were washed thrice with deionized water and treated with deionized water at 80 °C for 30 min, then washed three times with deionized water and dried at 150 °C for 30 min. The cleaned glass substrates were stored dry at room temperature.

### 2.3. Preparation of SnCl_2_-Modified Glass Substrates

A series of experiments were carried out to optimize glass modification with SnCl_2_. A set of SnCl_2_ aqueous solutions at concentrations ranging from 0.1% to 7.0% (*w*/*v*) were prepared by dissolving SnCl_2_·2H_2_O in 25 mL of deionized water. The cleaned glass substrates were immersed in the SnCl_2_ solution in a polypropylene tube and kept for 15–75 min at 25–100 °C. The substrates were then washed five times with deionized water and dried at 25–200 °C for 15 min (all varied conditions are shown in Table 1). The SnCl_2_-modified glass substrates were kept dry at room temperature.

### 2.4. Characterization of SnCl_2_-Modified Glass Substrates Using X-Ray Photoelectron Spectroscopy

X-ray photoelectron spectroscopy (XPS) characterization of the SnCl_2_-modified glass substrates was carried out using a photoelectron spectrometer, “KRATOS AXIS ULTRA DLD” (Kratos Co. Ltd., Manchester, England), with a spherical analyzer and X-ray source. Ion etching was not used. XPS was carried out at a hybrid lens mode with the following parameters: resolution: a pass energy of 40; acquisition time: 1102 s; anode: Mg (50 W); step: 200 meV for survey spectra and 100 meV for regions; and dwell time: 100 ms, with charge neutralizer switched on. The spectra were calibrated to the C 1 s signal (284.7 eV).

### 2.5. Spectral-Phase Interferometry

In this study, a new generation of portable SPI biosensors was used. They featured high energy efficiency (powered via a USB port from a personal computer) and enabled highly sensitive real-time measurements of biolayer thickness changes on the sensor chip surface [39,50]. The SPI technique is based on the use of a thin (100 µm) plane-parallel glass substrate as a two-beam interferometer illuminated by broadband low-coherent radiation from a superluminescent diode (Figure S1) [38]. The substrate serves, simultaneously, as a sensor chip for the investigation of intermolecular interactions. The interference between the reference beam reflected from the substrate surface in contact with the air and the probe beam reflected from the “liquid–glass surface with biomolecules” interface is used to monitor the dynamics of changes in the biolayer thickness on the surface. The result of the interference depends on the phase thickness of the combined glass and biomolecule layer. Its change is recorded by the phase shift in the interference pattern.

### 2.6. Ovalbumin Immobilization onto SnCl_2_-Modified GLASS Substrates

The SnCl_2_-modified glass substrates were placed in a flow cell of the SPI biosensor and washed with PBS buffer solution for 10 min at a flow rate of 1.7 µL/min. Then, 20 µL of OVA solution (50 μg/mL) in PBS was added into the SPI flow cell, and the change in signal was measured. The thickness of the adsorbed OVA layer averaged over the sensor chip surface was considered a reference.

### 2.7. Detection of AFB1 with Spectral-Phase Interferometry and Sensor Chips Based on SnCl_2_-Modified Glass Substrates

Similarly, the SnCl_2_-modified glass substrates were washed with PBS buffer inside the SPI biosensor. Then, 20 µL of an OVA-AFB1 conjugate in PBS solution (50 μg/mL) was injected into the SPI flow cell. To block the surface and remove unbound OVA-AFB1, a solution of BSA in PBS (1 mg/mL) was then added. Subsequently, 50 µL of a mixture of 40 µL of the analyzed solution containing AFB1, 5 µL of monoclonal antibodies against AFB1 (0.5 mg/mL), and 5 µL of 1% BSA in PBS was pumped through the biosensor flow cell. The calibration curve was fitted using a five-parameter logistic (5PL) function; the fitting parameters are listed in Table S1.

### 2.8. Extracting AFB1 from Corn Flour Samples

For the extraction of AFB1 from real food samples, 2.5 mL of acetonitrile was added to 1 g of corn flour. The mixture was intensively shaken for 15 min at room temperature. Then, the mixture was centrifuged at 4000 rcf for 10 min, and the supernatant was separated. The obtained extract was filtered through a filter with a 0.45 µm pore size to remove residual solid particles. Then, 900 µL of PBS solution containing 0.1% BSA was added to 100 µL of the purified extract, and the obtained sample was analyzed by spectral-phase interferometry using the sensor chips based on the SnCl_2_-modified glass substrates.

## 3. Results and Discussion

### 3.1. XPS Characterization of the Glass Sensor Chips Based on the SnCl_2_-Modified Glass Substrates

**Figure 1:**
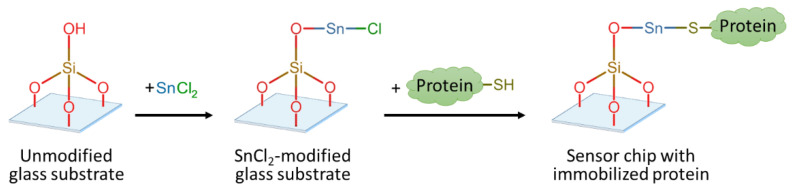
The proposed mechanism for the SnCl_2_ modification of glass substrates followed by protein immobilization.

The sensor chips developed in this work based on the SnCl_2_-modified glass substrates were used for a competitive assay for AFB1 detection by the label-free interferometric immunosensors. First, the glass surface was modified with SnCl_2_ for the further immobilization of a carrier protein to create a biofunctionalized surface for biosensing. The proposed mechanism of protein binding to the SnCl_2_-modified glass substrates is shown in Figure 1. As the first step, –SnCl groups were introduced onto the purified surface of borosilicate glass, followed by the immobilization of the carrier protein using strong Sn-S bonds due to the interaction of sulfur-containing amino acids that formed complexes with tin [51]. To confirm the glass modification by SnCl_2_, XPS spectra were obtained and analyzed for the following samples: (i) unmodified glass substrates after purification, (ii) SnCl_2_-modified glass substrates, and (iii) “fresh” and (iv) “aged” (3 days in air) sensor chips based on the SnCl_2_-modified glass substrates with the immobilized carrier protein.

**Figure 2:**
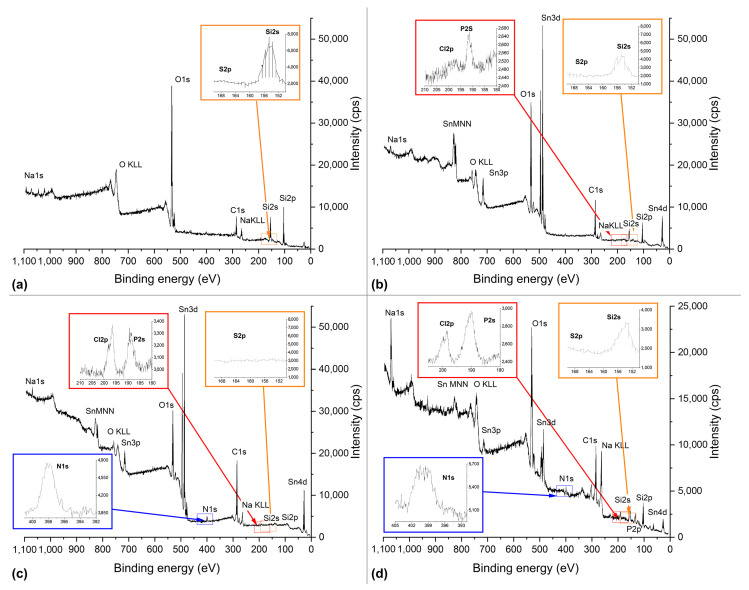
XPS spectra of the unmodified glass substrate after the purification of (**a**) the SnCl_2_-modified glass substrate (**b**) and the SnCl_2_-modified glass substrate with the immobilized carrier protein (OVA): “fresh” (**c**) and “aged” (**d**). The following parameters were used for the modification: SnCl_2_ concentration—2% (*w*/*v*); temperature of modification solution—50 °C; reaction duration—45 min; temperature of drying—150 °C.

The XPS spectra obtained for the unmodified glass substrates (Figure 2a) demonstrate Na1s (1070–1020 eV), Si2s (156–148 eV), and O1s (535–526 eV) peaks as expected for glass. No other significant peaks are evident. This indicates successful glass purification. The SnCl_2_ modification of the glass substrates causes additional peaks of Sn3d (496–481 eV) and Cl2p (190–193 eV), which indicate the formation of a Sn-containing layer with a small amount of Cl (Figure 2b). This confirms the successful modification of glass with SnCl_2_. The SnCl_2_-modified glass was then brought into contact with an OVA solution and washed with PBS to remove excess OVA from the glass surface. The resulting XPS spectra (Figure 2c) demonstrate P2s (193–185 eV) and N1s (400–395 eV) peaks attributed to the buffer and OVA, respectively. This indicates successful OVA immobilization on the modified glass.

To assess the effect of SnCl_2_-modified surface aging on protein binding, OVA was also immobilized on the “aged” SnCl_2_-modified glass, which was stored before for 3 days in air. The absolute value of the N1s peak for the “aged” modified glass substrates (Figure 2d) is lower than that for the “fresh” one (Figure 2c). The decreased XPS intensity of the Sn3d and Sn3p peaks in the “aged“ sample compared to the fresh SnCl_2_-modified glass substrate is likely due to the denaturation of the immobilized protein over time, which could cause a change in its distribution on the surface, further shielding the tin atoms and attenuating the tin signals in the XPS measurements. Additionally, the decrease in the Sn3d and Sn3p peaks may be due to hydrolysis in air, which could alter the correlation between Sn and O. Furthermore, the overall peak intensities in the “aged“ sample spectra are lower, which may correspond to relatively high charging of the “aged” sample due to its high resistance, thus complicating the process of obtaining accurate spectra. The S2p (155–170 eV (161.5 eV; 162.7 eV)), -S-S-, and -SH (161–166 eV) (163.5 eV; 164.4 eV) peaks are absent in all the XPS spectra obtained for the clean, chlorostannated, and protein-modified glasses. This may be due to the low quantity and complicated surroundings of sulfur in the structure of the immobilized protein. Additionally, an increase in the intensities of the Sn3d and Sn3p peaks on the “fresh” sample suggests better binding of OVA to the “fresh” modified glass substrates. This correlation between protein binding and Sn content on the glass surface supports the assumption that the protein binds with Sn on the modified glass substrate.

### 3.2. Optimization of the Procedure for SnCl_2_ Modification of Glass Substrates

The process of obtaining SnCl_2_-modified glass substrates was optimized by varying several parameters: the concentration of SnCl_2_, the temperature of the SnCl_2_ solution during glass modification, the duration of glass modification, and the drying temperature of the glass substrate after SnCl_2_ modification. The modification effectiveness was evaluated using a label-free SPI biosensor. For this purpose, a solution of the OVA carrier protein was passed along the surface of the modified glass, and the growth of the biolayer due to OVA binding to the modified surface was recorded. A greater increase in the biolayer thickness at this stage corresponded to higher density of OVA adsorption and was interpreted as higher modification efficiency.

**Figure 3:**
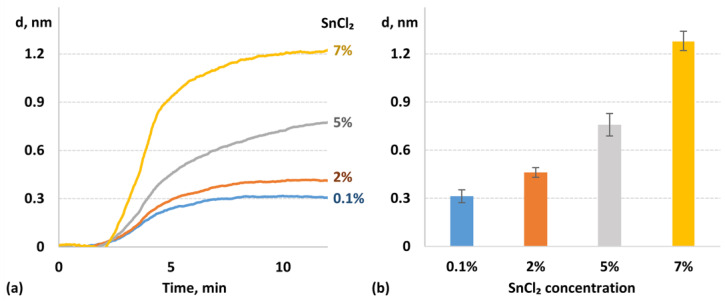
SPI sensograms indicating the real-time change in the biolayer thickness of OVA on the SnCl_2_-modified glass substrates at various SnCl_2_ concentrations (**a**) and the resulting total growth of the biolayer thickness (**b**). The following other parameters were used for the modification: temperature of modification solution—50 °C; reaction duration—45 min; temperature of drying—150 °C.

First, the concentration of the SnCl_2_ solution used for glass modification was varied from 0.1% to 7% (*w*/*v*) (Figure 3). At low SnCl_2_ concentrations, the growth of the OVA layer was minimal due to insufficient modification (Figure 3a). The layer thickness increased, with the SnCl_2_ concentration reaching a maximum of ~1.2 nm. We observed intensive hydrolysis of SnCl_2_ as its aqueous concentration increased. After modification with a 5% (*w*/*v*) solution, the SnCl_2_-modified glass was transparent. However, upon modification with a 7% (*w*/*v*) solution, extensive washing was required to remove the hydrolysis products of SnCl_2_ from the glass. It appears that at 5% (*w*/*v*) SnCl_2_, the hydrolysis products primarily interact with glass, leading to efficient modification, whereas at 7% (*w*/*v*) SnCl_2_, the hydrolysis products interact with both each other and the glass.

**Figure 4:**
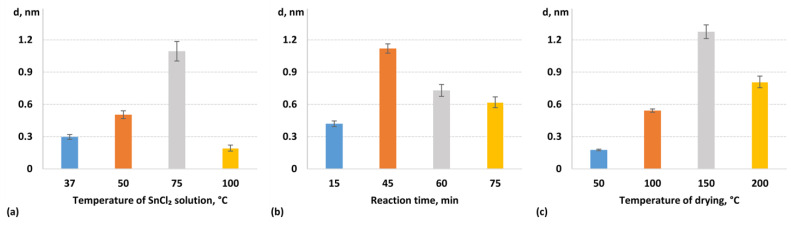
Optimization of the procedure for SnCl_2_-modified glass substrate preparation: effects on OVA layer thickness during the immobilization of SnCl_2_, with solution temperature (**a**), reaction time (**b**), and drying temperature (**c**) recorded using an SPI biosensor. SnCl_2_ concentration was 5% (*w*/*v*).

In the next series of experiments, we varied the temperature of the SnCl_2_ solution during glass modification. It was found that within the temperature range of 37–75 °C, the thickness of the immobilized OVA layer increased with the temperature of the SnCl_2_ solution (Figure 4a). This may be due to there being higher reaction rates at higher temperatures, as well as a higher hydrolysis rate, leading to more effective modification. However, at 100 °C, the layer thickness significantly reduced, which indicated unsuccessful glass modification with SnCl_2_. This could be due to the occurrence of an excessively high hydrolysis rate at higher temperatures, which may cause a loss of SnCl_2_ activity towards glass. The greatest thickness of the OVA layer was observed at 75 °C. Therefore, this temperature offers an optimal balance between the reaction rate and SnCl_2_ hydrolysis.

Similarly, it was experimentally found that the OVA layer thickness shows a bell-shaped dependence on the duration of the SnCl_2_-modification reaction (Figure 4b). At short reaction durations (15 min), the modification of the glass substrate was insufficient, while at longer durations (60–75 min), the SnCl_2_-modified glass substrate surface became opaque and white due to the high SnCl_2_ hydrolysis rate. The results of optimizing the drying temperature examined in the range of 50 to 200 °C are shown in Figure 4c. It was found that a drying temperature of 150 °C provides optimal effectiveness for SnCl_2_ modification.

Thus, these optimization studies revealed that the most effective SnCl_2_ modification conditions are as follows: 5% (*w*/*v*) SnCl_2_ solution at 75 °C for 45 min followed by drying at 150 °C. These conditions provided the best balance between modification efficiency and process control to obtain transparent and well-coated SnCl_2_-modified glass substrates, which were used in subsequent biosensing experiments for AFB1 detection.

Notably, one of the most common approaches to glass surface biofunctionalization with proteins involves the use of epoxy substrates. Immobilization occurs through covalent binding between the amino groups of the protein and the epoxy groups on the substrate. However, the epoxylation of glass surfaces requires the use of relatively expensive reagents like glycidoxypropyltrimethoxysilane (GOPTS), which is prone to hydrolysis upon exposure to air. This poses significant challenges in achieving predictable stability for both the GOPTS and the epoxylated glass. As a result, careful control over both the fabrication process and storage in a dry, inert atmosphere is necessary. An alternative to epoxylated substrates is aminated ones, which can also be used for immobilization via electrostatic interactions or by activating proteins with carbodiimides. However, these techniques are not as widely used for protein sensor chip fabrication. Electrostatic interactions often result in insufficiently stable bonds, while carbodiimide activation may lead to unwanted protein cross-linking. A more common approach involves the creation of aldehyde-functionalized substrates based on aminated glass. Nonetheless, this fabrication process involves multiple steps and requires quality control at each stage. Additionally, for certain applications, covalent immobilization involving the protein’s amine group may be undesirable, particularly when the functional region of interest contains a free amine group. In this context, the cost-effective, reproducible, and stable technique proposed in this work offers an efficient alternative to amine-targeted biomolecule immobilization, making it highly suitable for biosensor applications.

### 3.3. Determination of Aflatoxin B1 with Spectral-Phase Interferometry and Sensor Chips Based on the SnCl_2_-Modified Glass Substrates

**Figure 5:**
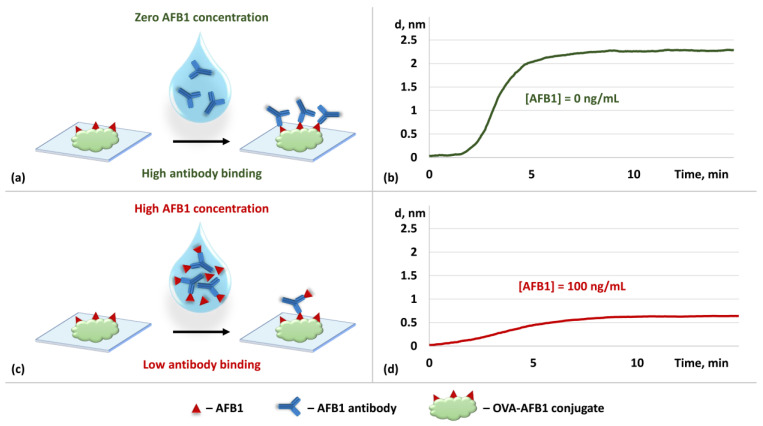
Competitive immunoassay for the detection of aflatoxin B1: schematic of the assay in the absence (**a**) and presence (**c**) of AFB1 in the sample at a concentration of 100 ng/mL and the corresponding experimental sensograms recorded using spectral-phase interferometry (**b**,**d**).

The optimized immobilization protocol for the SnCl_2_-modified glass substrates was used to implement a competitive immunoassay for AFB1 detection using spectral-phase interferometry. For this purpose, monoclonal antibodies capable of specifically recognizing AFB1 were added to the analyzed sample. Then, the analyzed sample was pumped along the SnCl_2_-modified glass substrates, which had OVA-AFB1 conjugates pre-immobilized on their surfaces (Figure 5a). If no AFB1 was present in the analyzed sample, the recognition antibodies bound to AFB1 molecules in the conjugate immobilized on the surface, thus producing an increase in the biolayer that was observable to the SPI sensor (Figure 5b). In the presence of the target toxin in the sample, some antibodies first bound to the AFB1 molecules in the sample (Figure 5c) and made some of the antigen-recognizing sites of the antibodies occupied. This decreased the capacity of these antibodies to bind to the AFB1 molecules on the sensor chip surface and reduced the observed biolayer growth. Figure 5d shows a characteristic SPI sensogram obtained in the experiments on analyzing the samples with AFB1 concentrations of 100 ng/mL.

**Figure 6:**
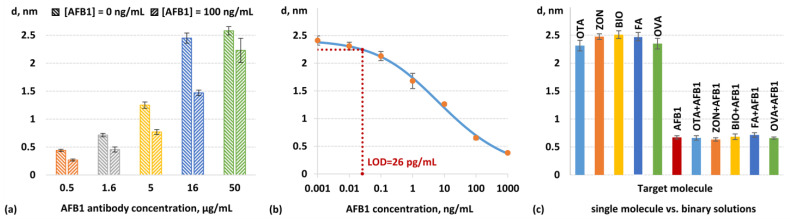
Immunoassay for AFB1 detection using sensor chips based on the SnCl_2_-modified glass substrates: optimization of monoclonal antibody concentration using SPI biosensor signals at 0 ng/mL and 100 ng/mL of AFB1—(**a**); calibration curve—(**b**); assay specificity in the presence of potentially interfering molecules: ochratoxin A (OTA), zearalenone (ZON), biotin (BIO), folic acid (FA), and ovalbumin (OVA) —(**c**).

The first step in the development of the immunoassay was the optimization of the monoclonal antibody concentration as the key factor that determines the sensitivity of competitive immunoassays. In these experiments, the target parameter to optimize was the ratio of SPI signals corresponding to biolayer thickness variations while pumping the antibody–sample mixture in two cases: (i) the sample without AFB1 and (ii) the sample containing AFB1 at a relatively low concentration of 0.1 ng/mL. Antibody concentrations of 0.5, 1.6, 5, 16, and 50 µg/mL were tested. As shown in Figure 6a, an increase in the antibody concentration in the range of 0.5–16 µg/mL elevates both the SPI signals and the optimized signal ratio. Meanwhile, at a concentration of 50 µg/mL, this ratio decreases, probably due to an insufficient amount of AFB1 in the sample to block the same proportion of antibodies at this high antibody concentration. Therefore, an antibody concentration of 16 µg/mL was selected for further experiments.

Figure 6b shows a calibration curve of the dependence of biolayer thickness growth on AFB1 concentration in a sample while the antibody–sample mixture is being pumped. As shown in Figure 6b, the experimental detection limit lies between measured concentrations of 0.01 ng/mL and 0.1 ng/mL. For the quantitative LOD, we used the 3σ criterion, which yielded an analytical LOD of 26 pg/mL. This dynamic range covers approximately five orders of concentration magnitude. Thus, the developed assay is comparable in sensitivity to HPLC-MS-based methods, and in terms of overall analytical performance, it surpasses modern test systems for AFB1 detection and HPLC-MS methods (Table 2). In combination with the advantages of label-free detection, the proposed method for the SnCl_2_ modification of glass substrates can be considered a promising platform for highly sensitive immunochemical systems.

To verify the specificity of the developed assay, we analyzed samples containing relatively high concentrations (100 ng/nL) of potentially interfering molecules, including other mycotoxins: ochratoxin A, zearalenone, biotin, folic acid, and ovalbumin. As can be seen in Figure 6c, the developed biosensor response was registered only for AFB1, whereas the potentially interfering molecules mentioned before did not contribute to the detected signal (both in the case of their single detection and in the study of binary solutions in the presence of AFB1). The response to OTA, ZON, BIO, FA, and OVA was 96%, 103%, 104%, 103%, and 98% of the signal corresponding to the blank sample, respectively. We also evaluated reproducibility by measuring 12 samples at a AFB1 concentration of 100 ng/mL. The relative standard deviation of the signal from the mean was calculated to be 6%, indicating the good reproducibility of the measurements. To validate the efficiency of the developed method for AFB1 detection in real samples, two corn flour samples previously characterized by HPLC-MS were analyzed. According to the HPLC-MS data, AFB1 was not detected in the first sample, while the second sample contaminated with *Aspergillus flavus* contained AFB1 at a concentration of 2.1 ng/mL. When these samples were analyzed using the developed SPI-based method with SnCl_2_-modified glass substrates, similar results were obtained. In the first sample, the AFB1 concentration was below the LOD, confirming the absence of contamination. In the second sample, the AFB1 concentration was 1.9 ng/mL, which corresponded to a recovery rate of over 90%. This high recovery rate highlights the accuracy and reliability of our biosensor for detecting AFB1 in real food samples and demonstrates its potential as an alternative to traditional techniques.

## 4. Conclusions

In this study, we successfully developed, characterized, and optimized SnCl_2_-modified glass substrates for the development of sensor chips for label-free interferometric biosensors for aflatoxin B1 detection. The SnCl_2_-modified glass substrates exhibited strong and stable immobilization of biomolecules through the interaction of tin (Sn) with sulfur-containing amino acids, leading to the formation of robust Sn-S bonds, as confirmed by the XPS analysis. The application of the optimized sensor chips in competitive immunoassays for AFB1 detection has demonstrated attractive analytical performance, including a detection limit of 26 pg/mL and a dynamic range of five orders of concentration magnitude. This method allowed for the accurate and reliable detection of AFB1 in complex matrices such as corn flour. That indicates its potential for real-world applications in food safety and agricultural monitoring.

Overall, the SnCl_2_-modified glass substrates developed in this study provide a promising platform for advanced label-free optical biosensors as a cost-effective, sensitive, and reproducible approach to AFB1 detection. Further efforts may focus on expanding this development to a broader range of analytes, including both contaminants and biomolecules, for environmental monitoring and medical diagnostics, as well as optimizing the scalability of this platform to create accessible, robust, and multi-target detection systems. Notably, the proposed approach not only provides a powerful tool for AFB1 detection for food safety monitoring but also demonstrates the potential of SnCl_2_-modified agents as versatile platforms for the development of next-generation biosensors, including both label-free and nanomaterial-based sensors [59,60,61].

## Figures and Tables

**Table 1:** Varied parameters of glass modification with SnCl_2_.

Parameter	Values			
SnCl_2_ concentration (*w*/*v*)	0.1%	2%	5%	7%
Temperature of modification solution	37 °C	50 °C	75 °C	100 °C
Reaction duration	15 min	45 min	60 min	75 min
Drying temperature	50 °C	100 °C	150 °C	200 °C

**Table 2:** Comparison of analytical characteristics of AFB1 detection with the proposed SPI-based method using SnCl_2_-modified glass substrates with typical immunoanalytical test systems and HPLC-MS methods.

Method	LOD, ng/mL	Dynamic Range, Orders	Assay Time	Ref.
**HPLC-MS**	0.16–0.32	3–4	10–60 min	[52,53]
**ELISA-based techniques**	0.62–8.61	2–4	2 h	[54,55]
**Lateral flow immunoassay**	0.06–1 ng/mL	2–4	6–30 min	[56,57,58]
**Spectral-phase interferometry**	0.026 ng/ml	5	15 min	This work

## Data Availability

Data are contained within the article.

## Supplementary Materials

The following supporting information can be downloaded at https://www.mdpi.com/article/10.3390/bios14110531/s1: Figure S1: Scheme of spectral-phase interferometry; Table S1: Calibration curve fitting parameters.
