# Supplementary material for: Stannous Chloride-Modified Glass Substrates for Biomolecule Immobilization: Development of Label-Free Interferometric Sensor Chips for Highly Sensitive Detection of Aflatoxin B1 in Corn
Source: Biosensors (Basel). 2024 Nov 3;14(11):531. doi: 10.3390/bios14110531 (PMC11591935; doi:10.3390/bios14110531)
Supplement: Supplementary file 1 [file biosensors-14-00531-s001.zip › biosensors-3244094-supplementary.pdf]

biosensors

MDPI

Supplementary Materials

Stannous Chloride-Modified Glass Substrates for Biomolecule Immobilization: Development of Label-Free Interferometric Sensor Chips for Highly Sensitive Detection of Aflatoxin B1 in Corn

Alexey V. Orlov [1,†], Maria O. Zolotova [1,2,†], Denis O. Novichikhin [1], Nikolai A. Belyakov [1,3], Svetlana G. Protasova [4], Petr I. Nikitin [1,*] and Artem V. Sinolits [1,2]

[1] Prokhorov General Physics Institute of the Russian Academy of Sciences, 38 Vavilov Street, Moscow 119991, Russia; alexey.orlov@kapella.gpi.ru (A.V.O.); zolotovamaria333@gmail.com (M.O.Z.); nammen@yandex.ru (D.O.N.); whitenik.bel@yandex.ru (N.A.B.); sinolits@geokhi.ru (A.V.S.)
[2] Vernadsky Institute of Geochemistry and Analytical Chemistry, Russian Academy of Sciences, Kosygin Str. 19, Moscow 119991, Russia
[3] Moscow Center for Advanced Studies, Kulakova Str. 20, Moscow 123592, Russia
[4] Osipyan Institute of Solid State Physics, Russian Academy of Sciences, Chernogolovka 142432, Russia; sveta@issp.ac.ru
* Correspondence: nikitin@kapella.gpi.ru
† These authors contributed equally to this work.

Citation: Orlov, A.V.; Zolotova, M.O.; Novichikhin, D.O.; Belyakov, N.A.; Protasova, S.G.; Nikitin, P.I.; Sinolits, A.V. Stannous Chloride-Modified Glass Substrates for Biomolecule Immobilization: Development of Label-Free Interferometric Sensor Chips for Highly Sensitive Detection of Aflatoxin B1 in Corn. *Biosensors* **2024**, *14*, 531. https://doi.org/10.3390/bios14110531

Received: 25 September 2024
Revised: 31 October 2024
Accepted: 1 November 2024
Published: 3 November 2024

Copyright: © 2024 by the authors. Licensee MDPI, Basel, Switzerland. This article is an open access article distributed under the terms and conditions of the Creative Commons Attribution (CC BY) license (https://creativecommons.org/licenses/by/4.0/).

1. Scheme of spectral-phase interferometry

The SPI technique is based on the use of a thin (100 μm) plane-parallel glass substrate as a two-beam interferometer illuminated by broadband low-coherent radiation from a superluminescent diode (Figure S1). The substrate serves, simultaneously, as a sensor chip for the investigation of intermolecular interactions. The interference between the reference beam reflected from the substrate surface in contact with the air and the probe beam reflected from the "liquid–glass surface with biomolecules" interface is used to monitor the dynamics of changes in the biolayer thickness on the surface. The result of the interference depends on the phase thickness of the combined glass and biomolecule layer. Its change is recorded by the phase shift in the interference pattern.

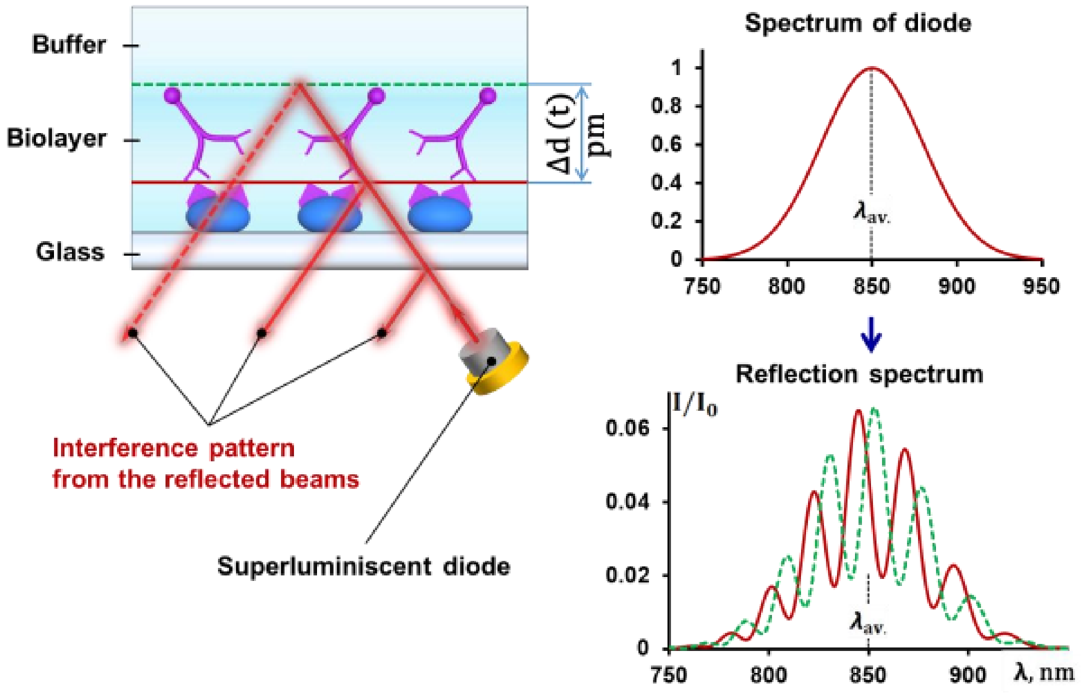

Figure S1. Scheme of spectral-phase interferometry.

2. Calibration curve fitting parameters

The experimental data were fitted using a five-parameter logistic (5PL) function. The equation is as follows:

$$S = d + \frac{(a - d)}{(1 + ([AFB1] \cdot c)^{b})^{m}}$$

where all parameters are described in Table S1.

Table S1. Calibration curve fitting parameters.

Parameter	Description	Value
S	The SPI response in nanometers	Dependent variable
[AFB1]	The concentration of aflatoxin B1 in ng/mL	Independent variable
a	The maximum signal	Fixed at 2.42 nm
d	The minimum signal	Fixed at 0 nm
c	Parameters determined by the least-squares method to minimize the difference between the curve and the experimental data	Calculated value: 0.385502656
b		Calculated value: 0.463404596
m		Calculated value: 0.670063583

Disclaimer/Publisher's Note: The statements, opinions and data contained in all publications are solely those of the individual author(s) and contributor(s) and not of MDPI and/or the editor(s). MDPI and/or the editor(s) disclaim responsibility for any injury to people or property resulting from any ideas, methods, instructions or products referred to in the content.
